# Expression of lipid metabolism-related genes in hepatocellular carcinoma and their prognostic significance

**DOI:** 10.12669/pjms.42.2.12774

**Published:** 2026-02

**Authors:** Jisen Zhao, Shujie Cheng, Yuanlong Zhou, Yang Yu

**Affiliations:** 1 Jisen Zhao Department of Hepatobiliary Surgery, Affiliated Hospital of Hebei University, Baoding 071000, Hebei, China; 2 Shujie Cheng Department of Hepatobiliary Surgery, Affiliated Hospital of Hebei University, Baoding 071000, Hebei, China; 3 Yuanlong Zhou Department of Hepatobiliary Surgery, Affiliated Hospital of Hebei University, Baoding 071000, Hebei, China; 4 Yang Yu Department of Hepatobiliary Surgery, Affiliated Hospital of Hebei University, Baoding 071000, Hebei, China

**Keywords:** Gene expression, Hepatocellular carcinoma, Lipid metabolism, Prognosis

## Abstract

**Objective:**

To investigate the expression profiles of key lipid metabolism-related genes in hepatocellular carcinoma(HCC) tissues, aiming to identify novel prognostic biomarkers for HCC.

**Methodology:**

This was a retrospective study. Sixty patients who underwent curative hepatectomy for HCC at the Affiliated Hospital of Hebei University between January 2022 to January 2025 were enrolled in this study. Immunohistochemistry was performed to assess the expression levels of SREBF1, FASN and ACLY in both HCC tissues and normal adjacent tissues(NATs). Expression differences between HCC tissues and NATs were compared. Clinical data were compared between the positive and negative groups. Kaplan-Meier survival curves were generated to evaluate overall survival (OS).

**Results:**

The positive expression rates of SREBF1, FASN and ACLY were significantly higher in HCC tissues compared with NATs(all *P <* 0.001). Compared with the SREBF1-negative group, the SREBF1-positive group had a higher proportion of tumors >5 cm and a lower proportion of CNCL stage Ia tumors(both *P<* 0.05). In the FASN-positive group, the proportion of poorly differentiated tumors and mean AFP level were significantly higher than in the FASN-negative group (both *P <* 0.05). Similarly, the ACLY-positive group had a significantly higher proportion of poorly differentiated tumors compared with the ACLY-negative group. Survival analysis showed significantly lower OS in the positive expression groups compared to their negative counterparts.

**Conclusion:**

The expression levels of the lipid metabolism-related genes SREBF1, FASN and ACLY are significantly elevated in HCC tissues compared with NATs. These genes are considered independent prognostic biomarkers in HCC.

## INTRODUCTION

Hepatocellular carcinoma (HCC) is the second leading cause of cancer-related mortality worldwide and its disease burden continues to rise. Epidemiological data indicate that of the approximately 700,000 new liver cancer cases diagnosed annually across the world, nearly 50% occur in China. In China, primary liver cancer ranks fourth in incidence and second in cancer-related mortality among all malignancies. Moreover, most cases are diagnosed at intermediate or advanced stages, resulting in poor overall survival (OS) rates.^1^-^3^ Although radical surgery offers a chance for long-term survival in patients with early-stage HCC, with five years survival rates of 80.5% for CNLC stage Ia, 47.7% for stage Ib and 37.2% for stage IIa, the recurrence rate remains high and significantly compromises long-term outcomes.^4^

In recent years, lipid metabolic reprogramming has been recognized as a critical driver of tumor development and progression. In HCC, the aberrant activation of lipogenesis pathways is closely associated with tumor cell proliferation, invasion and drug resistance.^5^,^6^ As a central regulator of lipid biosynthesis, sterol regulatory element-binding transcription factor 1(SREBF1) has been shown to promote lipid accumulation in tumor cells by upregulating downstream effectors such as fatty acid synthase (FASN) and ATP citrate lyase (ACLY), thereby facilitating malignant behaviors including proliferation, migration and chemoresistance.^7^

Although preliminary studies have explored the role of the SREBF1/FASN/ACLY axis in hepatocarcinogenesis, systematic investigations into their co-expression patterns and prognostic implications in HCC remain limited. Against this backdrop, this study focused on three pivotal lipid metabolism-related genes, namely SREBF1, FASN and ACLY and examined their expression profiles in resected HCC tissues. By integrating molecular expression data with clinical follow-up outcomes, we aimed to elucidate the prognostic significance of their co-expression and explore potential avenues for risk stratification and targeted therapeutic intervention in HCC.

## METHODOLOGY

This was a retrospective study. This study enrolled 60 patients with HCC who underwent curative hepatic resection at the Affiliated Hospital of Hebei University between January 2022 and January 2025. Clinical data collected from all enrolled patients included age, sex, tumor diameter, tumor number, liver function assessment (the Child-Pugh classification), China Liver Cancer (CNLC) staging, histological differentiation and serum AFP levels.

### Ethical approval:

The study was approved by the Institutional Ethics Committee of the Affiliated Hospital of Hebei University (No.:HDFYLL-KY-2024-198; date: November 07, 2024) and written informed consent was obtained from all participants.

### Inclusion criteria:

- Meeting the diagnostic criteria for HCC as outlined in the *Standard for Diagnosis and Treatment of Primary Liver Cancer*, confirmed by histopathological examination.^8^
- Undergoing curative resection at our hospital with available paired samples of fresh tumor and normal adjacent tissue (NAT) suitable for immunohistochemistry (IHC).
- An estimated life expectancy of ≥3 months.
- Aged 35-65.

### Exclusion criteria:

- Pathological diagnosis of metastatic liver cancer, cholangiocarcinoma, or hepatocellular-cholangiocarcinoma.
- Receipt of any form of antitumor therapy before surgery.
- Presence of other severe systemic diseases or malignancies.
- Coexisting severe fatty liver or hereditary metabolic liver diseases.
- Tissue specimens unsuitable for analysis due to RNA degradation or improper fixation.
- Malignant tumors in other sites of the body at the same time.

### Detection of SREBF1, FASN and ACLY Expression:

The expression levels of SREBF1, FASN and ACLY in HCC tissues and NATs were assessed using IHC. Postoperative tissue specimens were fixed in 4% paraformaldehyde at 4°C for 24 hour. After fixation, the tissues were washed three times with phosphate-buffered saline (PBS) for five minutes each to remove residual fixative. Specimens were then dehydrated through a graded ethanol series (each step lasting one hour), cleared twice in xylene (30 minutes per step) and embedded in paraffin to prepare tissue blocks. Paraffin-embedded blocks were sectioned at a thickness of 3-4 μm and the sections were baked at 60°C for two hours to enhance adhesion. Following conventional deparaffinization and hydration, antigen retrieval was performed using heat-induced epitope retrieval. Sections were placed in EDTA (ethylenediaminetetraacetic acid) buffer and heated in a microwave until boiling, then maintained at boiling temperature for 15-20 minutes, followed by cooling to room temperature. After thorough PBS washing, endogenous peroxidase activity was blocked with 3% hydrogen peroxide (H_2_O_2_) for 10-15 minutes at room temperature. Sections were then rinsed with PBS three times and incubated with a blocking serum solution for 20 minutes at room temperature.

Primary antibodies used were rabbit anti-human polyclonal antibodies against SREBF1, FASN and ACLY (all from Wuhan Fine Biotech Co., Ltd., China), diluted 1:100 in antibody diluent. The sections were incubated with the primary antibodies overnight at 4°C. After washing three times in PBS (five minutes each), sections were incubated with a secondary antibody working solution for 30 minutes at room temperature, followed by three additional PBS washes. DAB (diaminobenzidine) was used as the chromogen and color development was monitored under a light microscope. The reaction was terminated with distilled water upon adequate color development. Nuclei were counterstained with hematoxylin, followed by dehydration, clearing and mounting for microscopic evaluation.

### Interpretation of SREBF1, FASN and ACLY Expression:

All IHC sections were independently evaluated in a double-blind manner by two senior pathologists. Positive SREBF1 expression was localized in the nucleus, while FASN and ACLY positivity was confined to the cytoplasm. A semi-quantitative scoring system was used to assess expression based on staining intensity and the proportion of positively stained cells. Staining intensity was scored as follows: 0: no staining; 1: light yellow; 2: yellow-brown; 3: dark brown. The percentage of positive cells was scored as follows: 0: <5%; 1: 5-25%; 2: 26-50%; 3: 51-75%; 4: >75%. The final immunoreactive score (IRS) was calculated as: IRS = Staining intensity × Percentage score. An IRS ≥ 4 was considered positive expression. Based on the expression status of each gene, patients were stratified into SREBF1-negative and -positive groups, FASN-negative and -positive groups and ACLY-negative and -positive groups.

### Follow-Up:

Follow-up was conducted through outpatient visits and telephone interviews. The primary endpoint was OS, with the final follow-up date set at December 31, 2024.

### Statistical analysis:

Statistical analyses were performed using SPSS26.0. Continuous variables were tested for normality and homogeneity of variance; those meeting assumptions were expressed as mean ± standard deviation (*x̄±s*) and compared between groups using independent-samples t-tests. Categorical variables were expressed as frequencies and percentages (n[%]) and compared using the chi-square (χ^2^) test. Kaplan-Meier survival analysis was used to estimate OS and Cox regression analysis was employed to identify independent prognostic factors for OS. A P-value < 0.05 was considered statistically significant.

## RESULTS

The positive expression rates of SREBF1 (81.67% *vs*. 20.00%), FASN (76.67% *vs*. 20.00%) and ACLY (73.33% *vs*. 23.33%) were significantly higher in HCC tissues compared with NATs (all *P <* 0.01) (Table-I).

**Table-I:** Comparison of SREBF1, FASN and ACLY expression between HCC tissues and NATs.

Group	n	SREBF1-positive	FASN-positive	ACLY-positive
HCC tissues	60	49(81.67)	46(76.67%)	44(73.33)
NATs	60	12(20.00)	12(20.00)	14(23.33)
*χ^2^*		45.646	38.576	30.033
*P-value*		<0.001	<0.001	<0.001

In the SREBF1-positive group, the proportion of patients with tumor diameter >5 cm (71.43% *vs*. 27.27%) and those at CNLC stage Ia (16.33% *vs*. 63.64%) differed significantly compared with the SREBF1-negative group (both *P <* 0.05). In the FASN-positive group, the proportion of poorly differentiated tumors (50.00% *vs*. 14.29%) and the mean serum AFP level (487.36 ± 218.24 ng/mL *vs*. 312.85 ± 223.71 ng/mL) were significantly higher than in the FASN-negative group (*P <* 0.05, respectively). Similarly, the ACLY-positive group showed a significantly higher proportion of poorly differentiated tumors compared with the ACLY-negative group (54.55% *vs*. 6.25%, *P <* 0.05) (Table-II to IV).

**Table-II:** Association between SREBF1 expression and clinicopathological characteristics in patients with HCC.

Clinical Variable	SREBF1-positive group (n = 49)	SREBF1-negative group (n = 11)	χ^2^/t	P-value
Age (years, *χ̅*±*S*)	56.32±8.74	54.91±9.26	0.479	0.634
Sex (n[%])			0.003	0.960
Male	36(73.47)	8(72.73)		
Female	13(26.53)	3(27.27)		
Tumor diameter (cm, *χ̅*±*S*)			7.542	0.006
≤5 cm	14(28.57)	8(72.73)		
>5 cm	35(71.43)	3(27.27)		
Number of tumor(s) (n[%])			0.223	0.637
≤1	32(65.31)	8(72.73)		
>1	17(34.69)	3(27.27)		
Child-Pugh classification of liver function (n[%])			0.369	0.544
A	41(83.67)	10(90.91)		
B	8(16.33)	1(9.09)		
CNLC staging (n[%])			8.442	0.015
Ia	9(18.37)	6(54.55)		
Ib	25(51.02)	1(9.09)		
IIa	15(30.61)	4(36.36)		
Tumor differentiation level (n[%])			0.156	0.693
Poorly differentiated	21(42.86)	4(36.36)		
Moderately to well-differentiated	28(57.14)	7(63.64)		
AFP (ng/mL, *χ̅*±*S*)	423.51±286.37	387.64±254.19	0.382	0.704

**Table-III:** Association between FASN expression and clinicopathological characteristics in patients with HCC.

Clinical Variable	FASN-positive group (n = 46)	FASN-negative group (n = 14)	χ^2^/t	P-value
Age (years, *χ̅*±*S*)	55.82±8.91	56.43±9.05	0.224	0.824
Sex (n[%])			0.034	0.854
Male	34(73.91)	10(71.43)		
Female	12(26.09)	4(28.57)		
Tumor diameter (cm, *χ̅*±*S*)			0.331	0.565
≤5 cm	19(41.30)	7(50.00)		
>5 cm	27(58.70)	7(50.00)		
Number of tumor(s) (n[%])			1.165	0.281
≤1	29(63.04)	11(78.57)		
>1	17(36.96)	3(21.43)		
Child-Pugh classification of liver function (n[%])			0.007	0.932
A	39(84.78)	12(85.71)		
B	7(15.22)	2(14.29)		
CNLC staging (n[%])			1.851	0.396
Ia	10(21.74)	5(35.71)		
Ib	22(47.83)	4(28.57)		
IIa	14(30.43)	5(35.71)		
Tumor differentiation level (n[%])			5.633	0.018
Poorly differentiated	23(50.00)	2(14.29)		
Moderately to well-differentiated	23(50.00)	12(85.71)		
AFP (ng/mL, *χ̅*±*S*)	487.36±218.24	312.85±223.71	2.605	0.012

**Table-IV:** Association between FASN expression and clinicopathological characteristics in patients with HCC.

Clinical Variable	ACLY-positive group (n = 44)	ACLY-negative group (n = 16)	χ^2^/t	P-value
Age (years, *χ̅*±*S*)	56.18±8.83	55.31±9.42	0.332	0.741
Sex (n[%])			0.234	0.628
Male	33(75.00)	11(68.75)		
Female	11(25.00)	5(31.25)		
Tumor diameter (cm, *χ̅*±*S*)			0.302	0.582
≤5 cm	20(45.45)	6(37.50)		
>5 cm	24(54.55)	10(62.50)		
Number of tumor(s) (n[%])			0.043	0.836
≤1	29(65.91)	11(68.75)		
>1	15(34.09)	5(31.25)		
Child-Pugh classification of liver function (n[%])			0.107	0.734
A	37(84.09)	14(87.50)		
B	7(15.91)	2(12.50)		
CNLC staging (n[%])			2.100	0.350
Ia	9(20.45)	6(37.50)		
Ib	21(47.73)	5(31.25)		
IIa	14(31.82)	5(31.25)		
Tumor differentiation level (n[%])			11.260	0.001
Poorly differentiated	24(54.55)	1(6.25)		
Moderately to well-differentiated	20(45.45)	15(93.75)		
AFP (ng/mL, *χ̅*±*S*)	458.27±202.15	412.63±279.84	0.695	0.490

**Fig.1:**
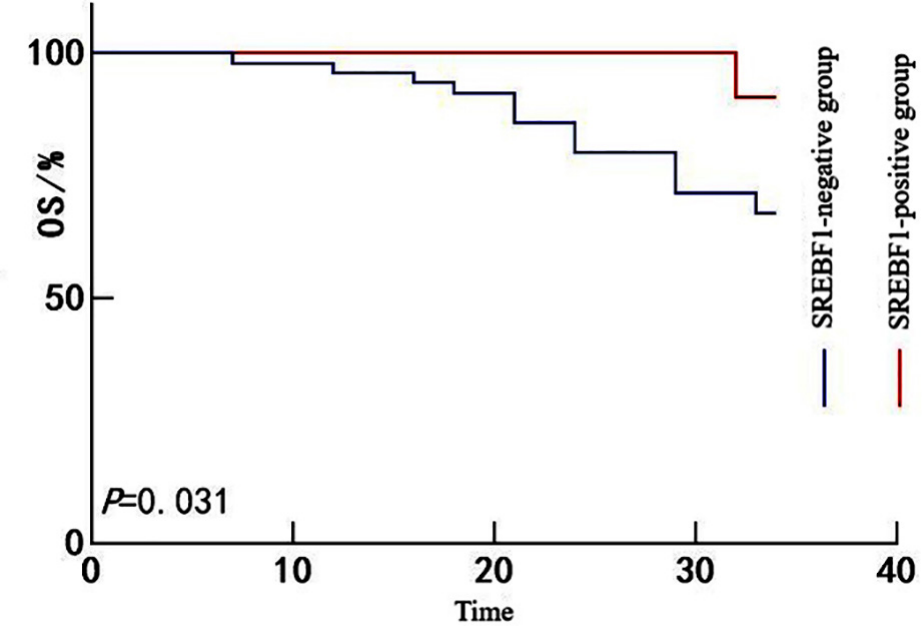
Kaplan-Meier survival curves for patients with HCC stratified by SREBF1 expression.

**Fig.2:**
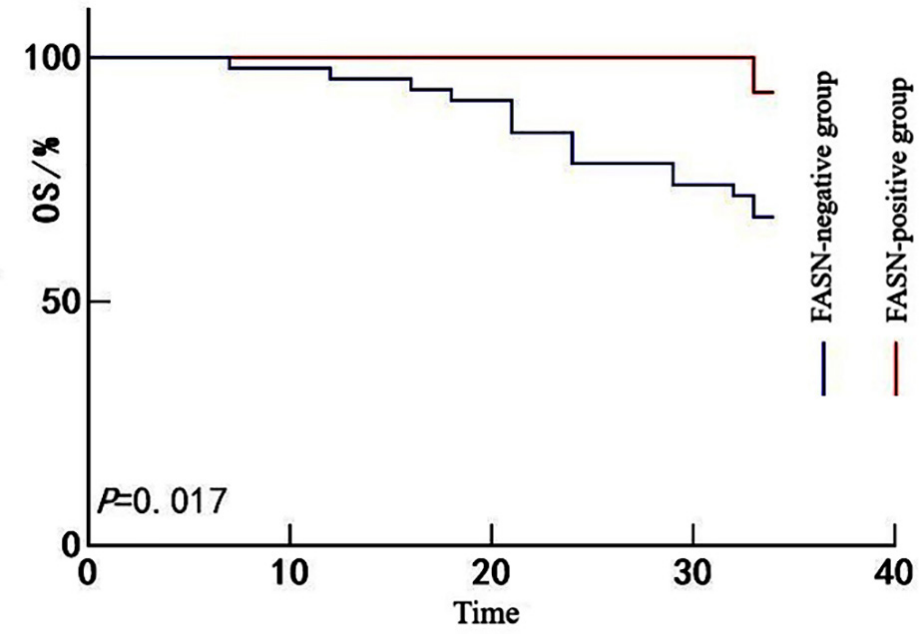
Kaplan-Meier survival curves for patients with HCC stratified by FASN expression.

**Fig.3:**
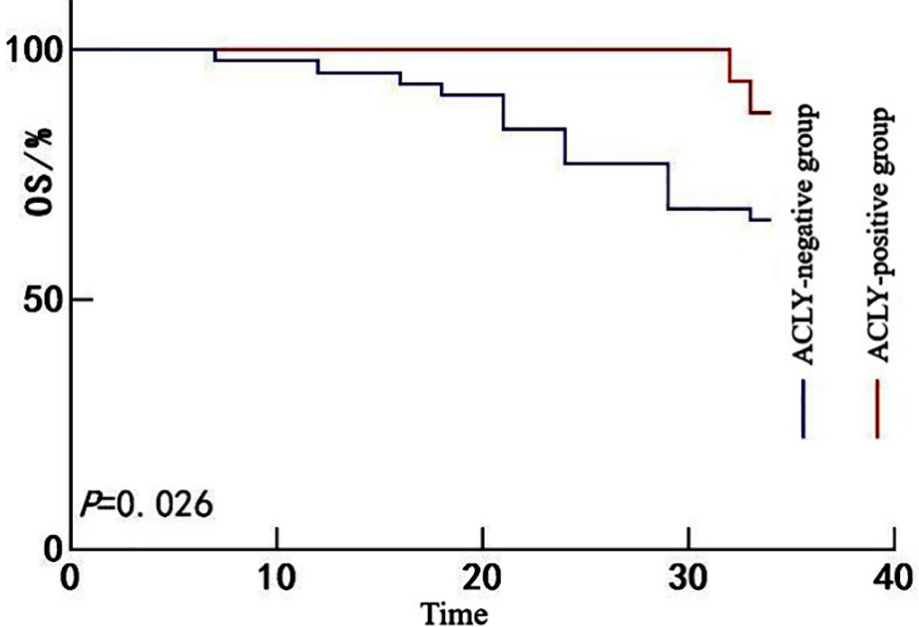
Kaplan-Meier survival curves for patients with HCC stratified by ACLY expression.

All 60 patients were followed for a period ranging from 6 to 34 months, with a median follow-up duration of 26 months. No patients were lost to follow-up. A total of 17 patients died during the follow-up period, yielding an OS rate of 78.33% (43/60). Kaplan-Meier survival analysis showed that the OS rate was significantly lower in the SREBF1-positive group than in the SREBF1-negative group (67.35% *vs*. 90.91%; Mantel-Cox χ^2^ = 4.677, *P =* 0.031) (Fig.1). Likewise, the OS rate in the FASN-positive group was significantly lower than in the FASN-negative group (67.39% *vs*. 92.86%; Mantel-Cox χ^2^ = 5.712, *P =* 0.017) (Fig.2) and the ACLY-positive group also exhibited poorer survival than the ACLY-negative group (65.91% *vs*. 87.50%; Mantel-Cox χ^2^ = 4.966, *P =* 0.026) (Fig.3).

Cox regression analysis revealed that the positive expression of SREBF1 (hazard ratio [HR] = 1.926, 95% confidence interval [95% CI]: 1.664-2.642), FASN (HR = 1.774, 95% CI: 1.326-2.687) and ACLY (HR = 1.683, 95% CI: 1.362-2.262) were independent risk factors for poor prognosis in patients with HCC (*P <* 0.001, respectively) (Table-V).

**Table-V:** Univariate and multivariate Cox regression analysis of prognostic factors in patients with HCC.

Variable	Univariate Analysis			Multivariate Analysis		
	HR	95%CI	P-value	HR	95%CI	P-value
Age (years)	0.902	0.826-1.065	0.412			
Sex	1.052	0.962-1.065	0.62			
Tumor diameter	1.604	1.261-1.899	0.007	1.125	0.919-1.313	0.082
Number of tumor(s)	1.145	0.891-1.188	0.298			
Child-Pugh classification of liver function	1.130	0.906-1.156	0.125			
CNLC staging	1.588	1.285-1.920	0.032	1.268	0.846-1.326	0.097
Tumor differentiation level	0.825	0.774-1.004	0.201			
AFP	1.147	0.907-1.205	0.475			
SREBF1-positive	1.828	1.323-2.535	<0.001	1.926	1.664-2.642	<0.001
FASN-positive	1.737	1.274-2.196	<0.001	1.774	1.326-2.687	<0.001
ACLY-positive	1.634	1.190-1.775	0.008	1.683	1.362-2.262	<0.001

## DISCUSSION

In our study, the expression level of SREBF1 was significantly higher in HCC tissues than in NATs, suggesting a potential oncogenic role of SREBF1 in hepatocarcinogenesis. As a central transcriptional driver of lipid metabolism, SREBF1 overexpression in HCC may reflect the abnormally heightened demand for lipid biosynthesis. In HCC, tumor cells achieve rapid proliferation through metabolic reprogramming and the de novo fatty acid synthesis directly reflects the upregulated expression of SREBF1.^9^,^10^ This metabolic adaptation not only provides tumor cells with sufficient phospholipids for membrane biosynthesis and ATP for energy but also contributes to resistance against oxidative stress and promotes mitochondrial remodeling, further enhancing tumor cell survival. The upregulation of SREBF1 may be driven by multiple upstream signaling cascades, particularly the mTOR and PI3K/AKT pathways. Existing literature shows that the activation of the mTOR and PI3K/AKT pathways can inhibit SREBF1 degradation; however, epigenetic modifications may relieve the transcriptional repression of SREBF1.^11^ Studies indicate that the aberrant activation of SREBF1 is closely associated with poor prognosis in HCC. Its overexpression not only sustains tumor metabolic demands through enhanced lipid metabolic reprogramming but also contributes to chemoresistance by modulating oxidative stress responses and mitochondrial function.^12^ The synergistic interaction between SREBF1 and its downstream targets FASN and ACLY may establish a positive feedback loop, intensifying lipid metabolic reprogramming and promoting tumor progression.

FASN is the only enzyme capable of catalyzing the de novo synthesis of long-chain saturated fatty acids using acetyl-CoA, malonyl-CoA and reduced nicotinamide adenine dinucleotide phosphate as substrates. This multifunctional homodimeric enzyme complex is predominantly expressed in the liver and adipose tissue under physiological conditions, where it facilitates the conversion of excess carbohydrates into fatty acids, which are subsequently esterified into triglycerides for storage and later metabolized via β-oxidation to generate energy.^13^ Our findings revealed that FASN was significantly overexpressed in HCC tissues compared with NATs, underscoring its crucial role in metabolic reprogramming and malignant progression in hepatocarcinogenesis. In the tumor microenvironment, rapidly proliferating cancer cells need substantial amounts of phospholipids for membrane synthesis and energy substrates for growth. FASN-mediated lipogenesis not only fulfills these metabolic demands but also enhances tumor cell survival by regulating oxidative stress tolerance and mitochondrial dynamics.^14^ This metabolic adaptation may be driven by the transcriptional activation and post-translational modification of FASN, primarily under the regulation of SREBF1. Such regulatory cascades lead to increased synthesis of long-chain fatty acids, such as palmitate, within HCC cells, thereby promoting aggressive tumor phenotypes. Previous studies have shown that the pharmacological inhibition of FASN reduces the half-maximal inhibitory concentration of sorafenib.^15^ These findings position FASN not only as a downstream effector of aberrant lipid metabolism in HCC but also as a central node connecting metabolic reprogramming, immune microenvironment disruption and therapeutic resistance.

ACLY functions as a core catalytic subunit in the de novo lipogenesis pathway by converting acetyl-CoA into malonyl-CoA, thereby supplying critical precursors for fatty acid biosynthesis.^16^ In our study, the positive expression rate of ACLY was significantly higher in HCC tissues than in NATs. ACLY plays a key role in metabolic regulation and energy homeostasis by linking glycolysis with lipid synthesis. Under oncogenic signaling stimulation, ACLY expression is amplified through both transcriptional activation by SREBF1 and epigenetic modifications, leading to intracellular accumulation of acetyl-CoA in HCC cells. This metabolic remodeling may be driven by oncogenic signaling cascades, epigenetic reprogramming and tumor microenvironmental cues, collectively promoting malignant progression.^17^ Previous studies have demonstrated that sorafenib-resistant HCC cells exhibit elevated lipid metabolic activity, along with increased ACLY expression. Genetic knockdown of ACLY not only effectively suppresses HCC cell proliferation, migration and invasion but also restores sorafenib sensitivity, particularly under hypoxic conditions.^18^,^19^

Our findings demonstrated that OS was significantly lower in patients with positive expression of SREBF1, FASN and ACLY compared with their respective negative expression groups. Multivariate Cox regression analysis further identified the positive expression of SREBF1, FASN and ACLY as independent prognostic risk factors for poor outcomes in patients with HCC. These results suggest that the three genes may serve as valuable molecular biomarkers for prognostic stratification in HCC. SREBF1, FASN and ACLY serve as central regulators of lipid metabolic pathways and their expression has been proven to be an independent risk factor for the prognosis of patients with HCC, providing an additional metabolic dimension to HCC prognostication.

While conventional staging systems such as TNM and CNLC primarily rely on tumor burden and hepatic functional reserve, integrating lipid metabolism-related gene expression into prognostic models could significantly enhance the sensitivity and precision of risk stratification in clinical practice. Moreover, for patients exhibiting high expression of SREBF1, FASN and ACLY, treatment strategies targeting lipid metabolism (*e.g*., combining sorafenib with lipid metabolism inhibitors) may offer improved antitumor efficacy.^20^ Although this study demonstrated that the expression levels of SREBF1, FASN and ACLY were significantly elevated in HCC tissues compared with NATs and further validated their prognostic value using Kaplan-Meier survival analysis and Cox regression models, several limitations should be acknowledged.

### Limitations:

First, the gene expression data were derived from a single-center cohort with a relatively small sample size and limited follow-up duration, which may have led to an underestimation of late recurrences. Second, the upstream and downstream regulatory networks of the SREBF1-FASN-ACLY axis were not experimentally validated in clinical specimens and the precise molecular mechanisms underlying their synergistic effects remain to be elucidated. Third, although the Cox regression model confirmed the individual prognostic significance of each gene, a quantitative risk stratification system incorporating these metabolic biomarkers was not established. Moreover, the study did not assess the potential of targeting lipid metabolism pathways to enhance sensitivity to adjuvant therapies.

## CONCLUSIONS

The positive expression rates of SREBF1, FASN and ACLY appear to be significantly higher in HCC tissues than in NATs. These genes are identified as independent risk factors for poor prognosis in HCC.

### Recommendations:

Future studies should consider multi-center, real-world cohorts with diverse etiological backgrounds to increase generalizability. Additionally, the development of an integrated prognostic scoring system that includes lipid metabolism-related genes may help further clarify the implications of these genes in HCC prognostication.

### Authors’ Contributions:

**JZ:** Was involved in the manuscript revision and validation, and was responsible and accountable for the accuracy or integrity of the work.

**SC:** Conceptualization, Writing - Review & Editing and Critical Review.

**YZ:** Methodology, Data Collection, Data Processing and Writing - Original Draft.

**YY:** Data Analysis & Interpretation.

All authors read and approved the final manuscript.

## References

[1] Xie D, Shi J, Zhou J, Fan J, Gao Q (2023). Clinical practice guidelines and real-life practice in hepatocellular carcinoma: A Chinese perspective. Clin Mol Hepatol.

[2] Hao L, Li S, Ye F, Wang H, Zhong Y, Zhang X (2024). The current status and future of targeted-immune combination for hepatocellular carcinoma. Front Immunol.

[3] Qu J, Yang J, Chen M, Cui L, Wang T, Gao W (2019). MicroRNA-21 as a diagnostic marker for hepatocellular carcinoma: A systematic review and meta-analysis. Pak J Med Sci.

[4] Merath K, Tiwari A, Court C, Parikh A, Dillhoff M, Cloyd J (2023). Postoperative Liver Failure: Definitions, Risk factors, Prediction Models and Prevention Strategies. J Gastrointest Surg.

[5] Cai J, Chen T, Jiang Z, Yan J, Ye Z, Ruan Y (2023). Bulk and single-cell transcriptome profiling reveal extracellular matrix mechanical regulation of lipid metabolism reprograming through YAP/TEAD4/ACADL axis in hepatocellular carcinoma. Int J Biol Sci.

[6] Yang X, Gu C, Cai J, Li F, He X, Luo L (2024). Excessive SOX8 reprograms energy and iron metabolism to prime hepatocellular carcinoma for ferroptosis. Redox Biol.

[7] Su F, Koeberle A (2024). Regulation and targeting of SREBP-1 in hepatocellular carcinoma. Cancer Metastasis Rev.

[8] Zhou J, Sun H, Wang Z, Cong W, Zeng M, Zhou W (2023). Guidelines for the Diagnosis and Treatment of Primary Liver Cancer (2022 Edition). Liver Cancer.

[9] Du D, Liu C, Qin M, Zhang X, Xi T, Yuan S (2022). Metabolic dysregulation and emerging therapeutical targets for hepatocellular carcinoma. Acta Pharm Sin B.

[10] Xu Y, Zeng J, Liu K, Li D, Huang S, Fu S (2024). USP11 promotes lipogenesis and tumorigenesis by regulating SREBF1 stability in hepatocellular carcinoma. Cell Commun Signal.

[11] Yi J, Zhu J, Wu J, Thompson CB, Jiang X (2020). Oncogenic activation of PI3K-AKT-mTOR signaling suppresses ferroptosis via SREBP-mediated lipogenesis. Proc Natl Acad Sci U S A.

[12] Zhang X, Bai Y, Zhu W, Lv X, Pei W (2022). ApoM regulates PFKL through the transcription factor SREBF1 to inhibit the proliferation, migration and metastasis of liver cancer cells. Oncol Lett.

[13] Menendez JA, Cuyas E, Encinar JA, Vander Steen T, Verdura S, Llop-Hernandez A (2024). Fatty acid synthase (FASN) signalome: A molecular guide for precision oncology. Mol Oncol.

[14] Yang H, Li J, Niu Y, Zhou T, Zhang P, Liu Y (2025). Interactions between the metabolic reprogramming of liver cancer and tumor microenvironment. Front Immunol.

[15] Shueng PW, Chan HW, Lin WC, Kuo DY, Chuang HY (2022). Orlistat Resensitizes Sorafenib-Resistance in Hepatocellular Carcinoma Cells through Modulating Metabolism. Int J Mol Sci.

[16] Convertini P, Santarsiero A, Todisco S, Gilio M, Palazzo D, Pappalardo I (2023). ACLY as a modulator of liver cell functions and its role in Metabolic Dysfunction-Associated Steatohepatitis. J Transl Med.

[17] Velez BC, Petrella CP, DiSalvo KH, Cheng K, Kravtsov R, Krasniqi D (2023). Combined inhibition of ACLY and CDK4/6 reduces cancer cell growth and invasion. Oncol Rep.

[18] Sun H, Wang F, Huang Y, Wang J, Zhang L, Shen Y (2022). Targeted inhibition of ACLY expression to reverse the resistance of sorafenib in hepatocellular carcinoma. J Cancer.

[19] Han Q, Chen CA, Yang W, Liang D, Lv HW, Lv GS (2021). ATP-citrate lyase regulates stemness and metastasis in hepatocellular carcinoma via the Wnt/β-catenin signaling pathway. Hepatobiliary Pancreat Dis Int.

[20] Ma APY, Yeung CLS, Tey SK, Mao X, Wong SWK, Ng TH (2021). Suppression of ACADM-Mediated Fatty Acid Oxidation Promotes Hepatocellular Carcinoma via Aberrant CAV1/SREBP1 Signaling. Cancer Res.

